# Breakdown of the Blood-Ocular Barrier as a Strategy for the Systemic Use of Nanosystems

**DOI:** 10.3390/pharmaceutics4020252

**Published:** 2012-05-14

**Authors:** Marcelo L. Occhiutto, Fatima R. Freitas, Raul C. Maranhao, Vital P. Costa

**Affiliations:** 1 Heart Institute, Medical School Hospital, University of São Paulo, São Paulo 05403-000, Brazil; Email: marceloocchiutto@uol.com.br (M.L.O.); fatima.sousa@incor.usp.br (F.R.F.); ramarans@usp.br (R.C.M.); 2 Faculty of Pharmaceutical Science, University of São Paulo, São Paulo 05508-000, Brazil; 3 Department of Ophthalmology, University of Campinas, Campinas, São Paulo 13083-887, Brazil

**Keywords:** blood-ocular barrier, hypotony, nanotechnology, ocular drug delivery system, ocular inflammatory mediators, systemic route

## Abstract

Several drug delivery systems have been proposed to overcome physiological barriers, improving ocular bioavailability. Systemic routes are seldom used due to the blood-ocular barrier. Novel drug delivery systems based on nanotechnology techniques have been developed to overcome ocular physiological barriers. This non-systematic review suggests the utilization of a transitory blood-ocular breakdown to allow the access of drugs by nanotechnology drug delivery systems via the systemic route. We discuss the possible ways to cause the breakdown of the blood-ocular barrier: acute inflammation caused by intraocular surgery, induced ocular hypotony, and the use of inflammatory mediators. The suitability of use of the systemic route and its toxic effects are also discussed in this article.

## 1. Background

Most ocular diseases are treated with topical drug applications formulated as solutions, suspensions or ointments. Ophthalmic topical formulations bear some drawbacks related to poor ocular bioavailability due to the many anatomical and physiological barriers existing in the eye [1]. The oral and intravenous routes are seldom used in ophthalmology, because the blood ocular barrier impairs the achievement of satisfactory drug concentration in intraocular tissues [2,3].

**Figure 1:**
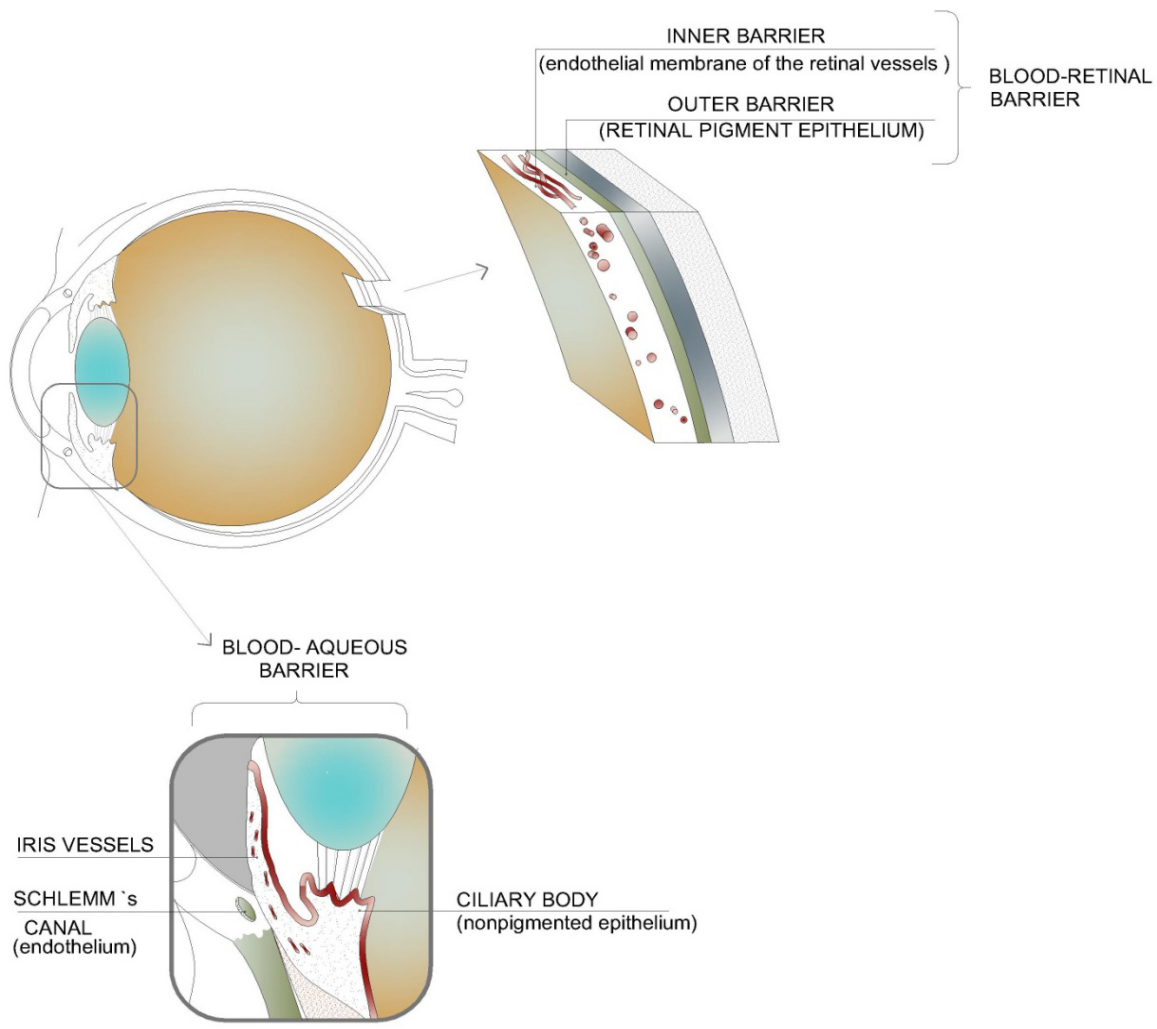
Blood-ocular barrier: blood-aqueous barrier and the blood-retinal barrier.

The blood ocular barrier is composed of the blood-aqueous barrier and the blood-retinal barrier, protecting the eye from entry of toxic substances and maintaining the homeostatic control that underpins the ocular physiology [4,5]. The blood-aqueous barrier is formed by the nonpigmented epithelium of the ciliary body, the posterior iris epithelium, the endothelium of the iris vessels with tight junctions of the leaky type, and the endothelium of Schlemm’s canal. The blood-retinal barrier consists of the retinal pigment epithelium (outer barrier) and the endothelial membrane of the retinal vessels (inner barrier), both with tight junctions of the nonleaky type. The two functional barriers restrict the movement of blood elements to the intraocular chambers [6] and explain why drugs administered orally or intravenously can hardly reach therapeutic levels in intraocular tissues (Figure 1). 

The barrier properties of the blood-brain barrier are similar to the blood-retinal barrier. Small protein tracers like microperoxidase (19 kDa) are capable of entering the perioxonal space but not the retina [6], indicating that the intercellular junctions of the retinal endothelium that are sealed with overabundant amounts of *zonulae occludentes* contribute to form the bulk of the blood-retinal barrier [7]. The ciliary epithelium of the ciliary body of the rabbit eye does not have as effective tight junctions as those found in the retina and in the brain capillaries [8]. Thus, the blood-aqueous barrier is not as active as the blood-retinal barrier in respect to limiting molecular diffusion. After intravenous injection, a lot of test substances, such as inulin, chloride, sucrose, phosphate, potassium, sodium, urea, proteins and some antibiotics could be found on the anterior side of the vitreous humor resulting from the ciliary circulation, but could not reach the retina due to the blood-retinal barrier [9].

**Scheme 1:**
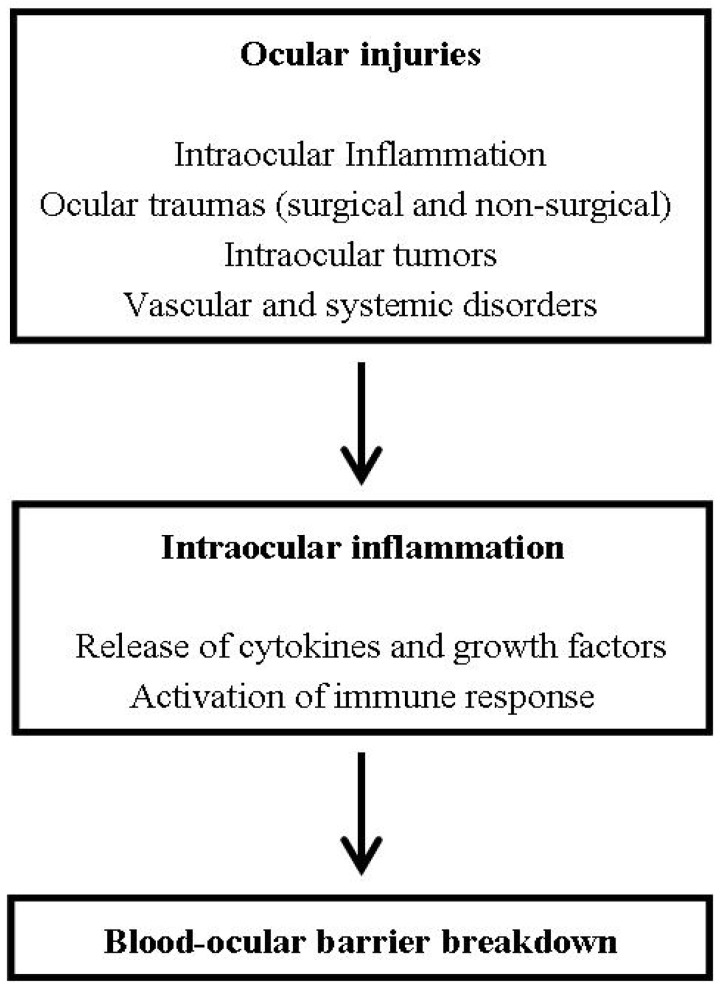
Effects of ocular injuries on the blood-ocular barrier.

There are some situations when the breakdown of the blood-ocular barrier can occur, such as ocular injuries and ocular hypotonia. Many types of ocular injuries, such as surgical and non-surgical traumas, intraocular inflammation (e.g., uveitis, scleritis), vascular disorders (e.g., M. Coats, M. Eales), systemic disorders (e.g., diabetes) and intraocular tumors (e.g., retinoblastoma, uveal melanoma) can disturb the blood-ocular barrier, resulting in a variable inward movement of inflammatory cells and blood plasma constituents such as proteins, cytokines and growth factors [10] (Scheme 1).

**Scheme 2:**
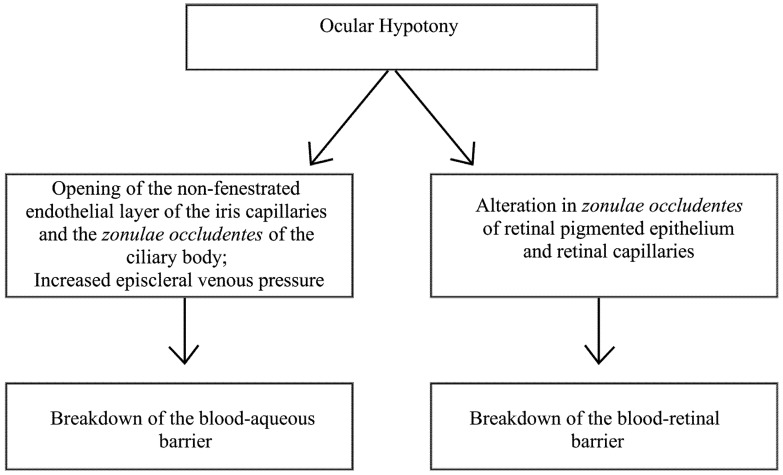
Effects of ocular hypotony on the blood-ocular barrier.

Ocular hypotony can promote the breakdown of the blood-aqueous barrier, permitting the leakage of serum proteins into the anterior and posterior chambers through the opening of the non-fenestrated endothelial layer of the iris capillaries and the *zonulae occludentes* of the ciliary body [11] (Figure 1). Ocular hypotony may also cause a breakdown of the blood-ocular barrier when intraocular pressure is lower than the episcleral venous pressure, leading to reversion of the flow direction of the ocular fluids, with plasma proteins entering the anterior chamber from the blood-filled Schlemm’s canal [12]. The blood retinal barrier is concomitantly broken down with the lowering of intraocular pressure, which affects the *zonulae occludentes* of the retinal pigmented epithelium (Verhoeff’s membrane) and the retinal capillaries [13,14,15] (Scheme 2).

The three commonly used ocular routes of drug administration are topical, local and systemic, but they bear limitations and may lead to complications. The topical route is frequently accompanied by toxicity in the cornea, conjunctiva and nasal mucosal [16,17,18,19]. Furthermore, daily topical instillation frequently leads to a lack of compliance [20,21]. Local toxicity can also develop with the use of subconjunctival and sub-Tenon routes. These procedures can cause muscle trauma [22], and inadvertent globe perforation [23,24]. Finally, retinal toxicity and infections may occur after intravitreal injections [25,26,27]. The systemic application of drugs is another method of reaching the posterior segment of the eye, but it is still a challenge due to the blood-ocular barrier. Despite the presence of an extensive vascular network in the choroid, the entry of drugs into the posterior segment of the eye is limited by the outer and inner blood-retinal barriers. In addition to the limitations imposed by the blood-ocular barrier, other facts limit the systemic application, since dilution and degradation of the drug before reaching the target site may occur. However, the systemic route could be a useful alternative, since this route is less invasive and the patients prefer it to the local route in the treatment of some diseases. There is the additional benefit of facilitating out-patient treatment [28].

Innovative techniques of drug delivery are required to overcome the limitations of systemic treatment of ophthalmic disorders. One of these strategies is the use of nanoparticles in drug delivery systems, that can be intravenously administrated and that overcome the physiological barriers.

## 2. Nanosystem

**Figure 2:**
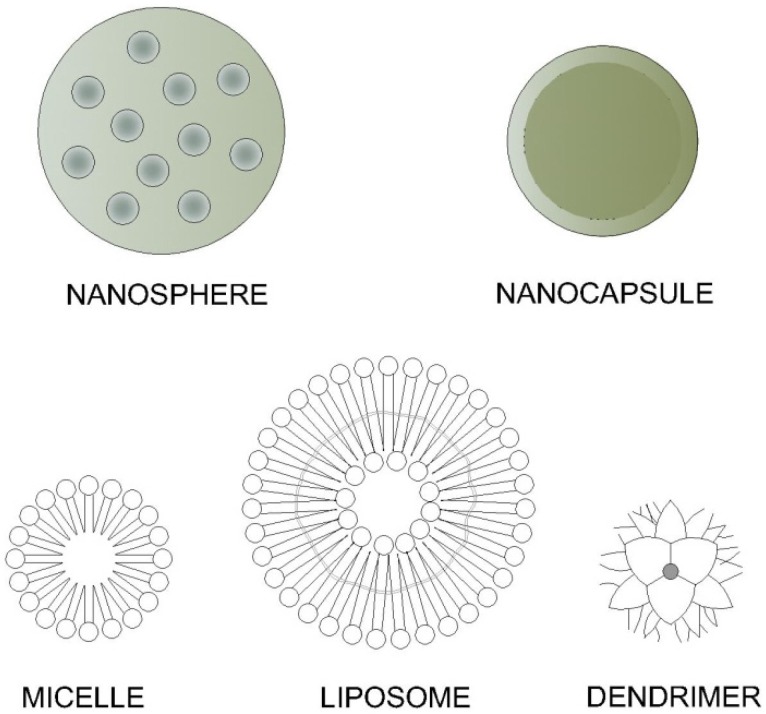
Examples of nanosystems [29].

Any physical system that is engineered at nanoscale is defined as a nanosystem. Nanosystems include nanoparticles (nanospheres and nanocapsules), liposomes, dendrimers, niosomes, and soluble macromolecules, amongst others. Some examples of these systems are shown in Figure 2.

Nanoparticles are spherical particles varying in size from 10 nm to 1 µm, containing a drug molecule or not. They can be prepared using materials of different chemical classes, for example proteins, lipids, polymers or polysaccharides [30]. The active agent may be encapsulated/entrapped, dissolved or distributed to a nanoparticle matrix, and then can be broadly classified into nanospheres, nanoparticles or nanocapsules. 

Nanoparticles can have the ability to overcome physiological barriers, passing even through capillaries, and transporting the drug to specific cells or intracellular compartments by several targeting mechanisms [31,32,33,34]. Singh *et al*. used arginine-glycide-aspartic acid peptide or dual-functionalized poly-(lactide-co-glycolide) nanoparticles to target delivery of anti-vascular endothelial growth factor (VEGF) intraceptor plasmid to choroidal neovascularization lesions in rats. They demonstrated that nanoparticles enable targeted gene delivery to the neovascular rat eye but not the control eye [35].

Liposomes are membrane-like vesicles that consist of one or more concentric phospholipid or cholesterol bilayers designed to carry a drug either into the core or into the bilayer. They can have a positive, negative or neutral surface charge, depending on their chemical composition, and they present different properties, such as binding affinity [36].

Light-targeted liposomal delivery was employed after systemic administration of thermosensitive liposomes for the diagnosis and therapy of chorioretinal neovascularization in age-related macular degeneration, showing the possibility of targeting a particular tissue [37]. In this approach, a liposomal formulation of a light-sensitive delivery system was selectively withheld in neovascular areas of the eye for the treatment of age-related macular degeneration, which demonstrated targeted therapy, even though this therapy was not completely successful in its purpose of destroying abnormal blood vessels without causing damage to normal surrounding tissues.

Dendrimers, macromolecular compounds from which a number of highly branched, tree-like arms originate in a symmetric fashion [38], are considered attractive for biomedical applications due to their unique physicochemical properties. As the dendrimer’s size can be controlled based on the stepwise chemical synthetic processes of their generation, they can become similar in size to a number of biological structures, such as G5 polyamidoamine dendrimers with the size of a hemoglobin molecule [39]

Niosomes are bilayered nonionic surfactant vesicles chemically stable with the ability to carry both lipophilic and hydrophilic drugs. Niosomes are nonimmunogenetic, biodegradable and biocompatible, and show low toxicity due to their nonionic structure [40].

Micelles are aggregates of amphiphilic molecules dispersed in a liquid colloid. Some micelar formulations for topical applications seem to be promising, but one of the major disadvantages of micellar formation is the demicellization that happens as a result of dilution upon injection *in vivo* [29].

Macromolecules in nanometer dimensions, when present in solution, including virtually any drug, can provide special properties to the drug molecule. Macugen^®^, a marketed treatment for neovascular age-related macular degeneration, is a nanoconjugate of an oligonucleotide (aptamer) with two polyethylene glycols (PEG) that prolongs the half-life of the drug in the vitreous humor [41].

Our laboratory has pioneered the use of artificially made, solid nanoparticles directed to therapeutic targets, called LDE [42]. This nanoemulsion has a structure similar to that of a low-density lipoprotein (LDL), with a core constituted of cholesteryl esters with trace amounts of triglycerides, surrounded by a phospholipid monolayer; at the surface monolayer there is a small proportion of free cholesterol. When the LDE is injected into the bloodstream, it acquires apo E from the native lipoproteins and binds to LDL receptors that recognize both apo B and apo E [42]. The mechanism behind this drug delivery system lies in the overexpression of cell surface receptors that bind and internalize LDL, as well as the artificial lipid nanoemulsions in tissues undergoing cell proliferation [43,44,45]. This drug-targeting nanoemulsion system can be used not only for cancer treatment, but also to carry drugs directed against atherosclerotic lesions in rabbits [46,47,48]. We also showed that the intense immunogenic-inflammatory processes in rabbits following heart transplantation could be attenuated when using nanoemulsion associated with anti-proliferative drugs [49].

### Applications of Nanosystems in Ophthalmology 

Constant efforts have been made toward the development of ocular drug delivery systems with various pharmacological properties, such as increased bioavailability, prolonged drug release, compatibility with ocular tissues, ease of use in the form of eye drops causing no blurred vision or irritation and fewer instillations to obtain the intended therapeutic effect. 

There are some interesting studies about topical applications of nanotechnological drug delivery systems for ophthalmic treatments. Adibkia *et al*. showed that piroxicam nanoparticles of Eudragit RS 100 lead to a better control of inflammatory symptoms compared with a microsuspension of piroxicam alone in rabbits with endotoxin-induced uveitis [50].

De La Fuente *et al*. have shown that a hyaluronic acid-chitosan nanoparticles system was successfull in transfection experiments for ocular gene therapy. The results demonstrated that bioadhesive polysaccharides, hyaluronic acid and chitosan nanoparticles were able to provide high transfection levels in cornea and conjunctiva, without affecting cell viability [51].

Ciclosporin-loaded nanoparticles are also used for the treatment of extraocular disorders, such as keratoconjuctivitis sicca. *In vivo* experiments showed that, following topical instillation of cyclosporin-A-loaded chitosan nanoparticles into rabbits, it was possible to achieve therapeutic concentrations in external ocular tissues (*i.e.*, cornea and conjunctiva) during at least 48 h while maintaining negligible cyclosporin-A levels in inner ocular structures. Additionally, the chitosan nanoparticles levels were significantly higher than those obtained following instillation of a chitosan solution containing cyclosporin A and an aqueous cyclosporin suspension [52]. Table 1 shows some other nanostructured drug delivery systems used in ophthalmology.

**Table 1:** Examples of nanotechnology based ocular drug delivery systems.

Active Agent	Drug Delivery System	References
Ibuprofen Ciclosporin Diclofenac sodium	Solid lipid nanoparticles	[53,54]
Ciclosporin Dexamethasone Pilocarpine	Nanoemulsion	[55,56]
Dexamethasone	Micelles	[57]
Flurbiprofen	Nanosuspension	[58]
Timolol maleate	Discosomes	[59]
Pilocarpine nitrate Dexamethasone	2-hydroxypropyl-â-cyclodextrin	[60,61]
Acetazolamide Inulin Oligonucleotides Pilocarpine hydrochloride Diclofenac sodium	Liposomes	[62,63]
Tropicamide Pilocarpine nitrate	Dendrimers	[64]
Cyclopentolate	Niosomes	[65]

Since studies about the use of nanosystems applied systemically for ocular permeation are still scarce, further studies on the development of nanosystems are necessary to accomplish the purpose of ocular delivery of the desired drug in an efficient and safe way. 

## 3. Systemic Administration

Despite the limitations of the systemic route to ocular drug delivery, researchers have made continuous efforts to develop drug delivery systems to administer drugs systemically in order to clear these barriers. 

Among the challenges of effective systemic nanodelivery into tissues, the limited penetration across the vascular endothelium and the uptake by the reticuloendothelial system are considerable factors. The current nanodelivery systems depend on transvascular exchange and tissue accumulation, which require high dosages to create large concentration gradients to drive nanoparticles passively across the blood-tissue interface [66]. Nevertheless, passive accumulation allows only a fractional dosage of nanoparticles penetrating into target tissues, which diminishes therapeutic efficacy and aggravates potential side effects. Therefore, active delivery of targeted nanoparticles across the vascular endothelium could increase the desired therapeutic index with fewer side effects [66]. In addition, other facts limit the systemic application, since dilution and degradation of the drug before reaching the target site can occur. Furthermore, drug-drug interactions in patients being treated for coexisting diseases may also influence the administration of systemic drugs [66].

The use of nanoparticles that have a higher selectivity for the target tissue is a challenge to be achieved to improve systemic administration. Hence, specific targeting systems are needed to transport molecules to deeper layers of the eye, overcoming the blood-ocular barrier. Recent studies have shown promising results with systemic administration of nanoparticles. Kim *et al*. have demonstrated that 20 nm gold nanoparticles, administered intravenously, were able to pass through the blood-retinal barrier and were distributed into all retinal layers [67]. The absence of toxicity was noted in retinal endothelial cells, astrocytes and retinoblastoma cells [67]. Another recent study demonstrates that transferrin, arginine-glycine-aspartic acid peptide, or dual-functionalized poly-(lactide-co-glycolide) nanoparticles carrying anti-VEGF intraceptor plasmid, administrated intravenously, were able to reach choroidal neovascularization lesions, probably due to the broken blood-retinal barrier as a result of choroidal neovascularization in the laser-treated rat eye [39].

The similarities between the blood-brain and the blood-retinal barriers allow the proposal of the hypothesis that there is the possibility of systemically administered nanoparticles also gaining the ability to pass through the ocular barrier. Dalargin, a hexapeptide analog of leucine-enkephalin containing D-alanine, which produces central nervous system analgesia, can cross the blood-brain barrier when conjugated with poly(butylcyanoacrylate) nanoparticles and accumulate in the brain of mice, but not when it administrated without nanoparticles [68]. Furthermore, these studies on blood-brain barriers suggest the future application of systemically administered nanosystems for ophthalmic purposes. 

### Kinetics of Systemically Administered Nanoparticles

The systemic use of nanoparticles in order to reach ocular tissues is attractive, but more accurate kinetics studies are needed to show the real possibility of their safety and effectiveness.

*In vitro* experiments should have some attributes to be accurate, such as the ability to measure pathological situations separately (e.g., oxidative stress and inflammation), and the possibility to represent cell types targeted by nanoparticles through common routes of exposure (e.g., macrophages and other elements of the reticuloendothelial system) [69,70].

*In vivo* biodistribution of nanoparticles is a new and wide field of research. Several studies have been published recently describing the nanoparticle distribution, but, even though the attempts to modify their features, such as size, shape, surface coating and dosing, aiming to enhance treatment effectiveness by extending the blood circulation, nonspecific distribution has thus far been unavoidable. Excretion of nanoparticles is a concern that needs further long-term studies, since a recent study showed the variation in the levels of nanoparticle excretion dependent on time. Lastly, there is no standardized characterization of possible interaction of nanoparticles with proteins and immune cells [29].

A list of some examples of pharmacokinetic experimental and clinical studies using nanoparticles is summarized in Table 2.

**Table 2:** Examples of pharmacokinetic nanoparticle *in vitro* and *in vivo* studies.

Author	Study Approach	Reference
Shokeen *et al*.	Radiolabeled nanoparticles with positron emitting radionuclides along *in vitro* and *in vivo* protocols.	[71]
Almeida *et al*.	Comprehensive review of *in vivo* biodistribution of nanoparticles, including structure, toxicity and *in vitro* comparison.	[72]
Khlebtsov *et al*.	Comprehensive review of *in vitro* and *in vivo* biodistribution of nanoparticles, with nanoparticle parameters, used doses and toxicity.	[73]
Pires *et al*.	Treatment of breast cancer using paclitaxel associated to an artificial lipid nanoemulsion, emphasizing the improved pharmacokinetic parameters and the reduced toxicity of the drug.	[74]
Maranhão *et al*.	Plasma kinetics and biodistribution of an artificial lipid nanoemulsion in patients with acute leukemia.	[43]
Azevedo *et al*.	Plasma kinetics and biodistribution of an artificial lipid nanoemulsion associated to etoposide oleate in patients with ovarian carcinoma.	[44]
Saha *et al*.	Review of preparation, characterization, biodistribution and pharmacokinetic of nanosystems for cancer chemotherapy.	[75]

## 4. Toxicity of Nanoparticle Systems

The evaluation of systemic nanosystem toxicity is a complex task, since the drug-delivery systems have to take into account the toxicity of the polymer used, the drug, and entire system after administration *in vivo*. Moreover, systemic use of nanoparticles can induce changes in every organ of the human body, which may cause alterations in blood clinical chemistry, liver function, kidney function, blood cell counts, and other unknown side effects. 

The establishment of a complete risk-benefit ratio of the use of nanoparticles by systemic routes depends on the system configuration, specific materials used, dosage, and frequency of administration, besides the individual patient factors. Any change in these elements can affect the immunological response, cellular uptake, excretion, and, by consequence, the toxic effects elicited by the nanoparticle. 

New methods and biological models are necessary to increase the research in nanotoxicology. Recent toxicology studies seem to be promising to reach new evaluation standards. Kong *et al*. have shown the most important factors affecting nanoparticle cytotoxicity assays due to the current lack of standardized protocols, in order to improve the method that could evaluate the toxicity without false-negative or false-positive misinterpretations [76].

Almeida *et al*. have recently published an extensive review about nanoparticle biodistribution and toxicity [72]. The authors pointed out that nanoparticle modifications can be made to prolong blood circulation and enhance treatment efficacy, but simultaneous nonspecific distribution is unavoidable, as well as the need for long-term *in vivo* studies to properly investigate the innate and adaptive immune response, excretion over time and toxicity of nanoparticles. Another important point of this study is the need for more systematic *in vitro* approaches to improve the correlation with *in vivo* work, and to clarify the real nanoparticle therapeutic action and side effects [72].

Stensberg *et al*. have shown the activity, transport and fate of silver nanoparticles at the cellular and organismic level, in conjunction with the available methods of nanoparticle characterization [77]. They proposed several mechanisms of cytotoxicity based on such studies, as well new opportunities for investigating the uptake and fate of silver nanoparticles in living systems [77].

Maranhão *et al*. have shown that the association of chemotherapeutic agents, such as carmustine, etoposide, and paclitaxel, with artificial lipid nanoemulsion (LDE) pronouncedly reduces the toxicity of those drugs while preserving or even increasing the antineoplastic activity, as shown in tumor-implanted rats and mice [78,79] and in trials enrolling patients with advanced cancers [43,44,45,74].

There are recent reports regarding the toxicity concerns of the use of nanoparticles for ophthalmic purposes. Escobar *et al*. have shown that budesonide-PLA microparticles administered periocularly in rabbits remained at the periocular site of injection for a long time without causing local side effects, such as elevation in intraocular pressure, lens opacity or blood chemistry changes [80]. In addition, Amrite *et al*. have demonstrated that periocular administered celecoxib-PLGA particles were useful sustained drug delivery systems for inhibiting diabetes-induced elevation in PGE2 and VEGF without inducing fibrotic reactions at the site of administration or damage to the retina [81]. 

The systemic use of nanoparticle drug delivery systems for ophthalmic treatments is rare, equally scarce are the corresponding toxicological studies regarding this route of administration.

## 5. Hypothesis

The breakdown of the blood-ocular barrier is an attractive manner to allow the penetration of systemically applied drugs into the eye. We hypothesize that the breakdown of the blood-ocular barrier—that may occur in acute inflammation following intraocular surgeries, induced ocular hypotony and controlled use of inflammatory mediators—could allow the use of drugs carried in targeting nanosystems administered by the systemic route for ophthalmic purposes.

## 6. Blood-Ocular Barrier Breakdown

Blood-ocular barrier breakdown can occur in some situation, such as acute inflammation caused by intraocular surgery, induced ocular hypotony, and the use of inflammatory mediators.

### 6.1. Ocular Surgeries

The majority of the intraocular surgical procedures may cause a breakdown in the blood-ocular barrier, leading to increased protein content in the aqueous humor and edema of the sensory retina, due to the induced inflammation [82]. The spectacular advances in intraocular microsurgery of both the anterior and posterior segment in recent years allow surgeons to perform minimally traumatic procedures. However, every surgical manipulation of the eye causes ocular inflammation and may lead to many adverse effects [83], if not inhibited by the use of anti-inflammatory medication.

The sequence of events that follow ocular trauma includes vasodilatation, increased blood flow and hyperemia, increased permeability of blood vessels, edema, increased tissue pressure (disrupted blood-ocular barrier), and the presence of inflammatory cells [82].

Almost all morphological, physiological and metabolic responses to trauma may be attributed to the synthesis and release of endogenous mediators, such as prostaglandins [84], leukotriens, and various cytokines such as interleukins, tumor necrosis factor α and VEGF [85], causing a breakdown of the blood-ocular barrier. The critical phase of inflammation caused by surgical trauma begins at the conclusion of surgery and continues up to the first postoperative week [83,86]. The subconjunctival corticosteroid injection at the end of the surgical procedure is the current practice [87]. Thus, theoretically, the correct time for the use of systemic medication, as an alternative to reduce ocular inflammation, would be at the conclusion of surgery, taking advantage of the breakdown of the blood-ocular barrier. 

### 6.2. Induced Ocular Hypotony

The mean intraocular pressure is described statistically to be around 15.9 ± 2.9 mmHg [88]. Therefore, hypotony could be defined as an intraocular pressure below two standard deviations from the mean. In recent years, most authors [89,90,91,92] have been considering an intraocular pressure of ≤5 mmHg as clinically relevant. However, using a single cut-off value to define hypotony may be inadequate, since low intraocular pressure can be completely asymptomatic, without causing any irreversible functional damage [93]. 

Transient hypotonia, when it is resolved in fifteen to thirty days, is relatively common in clinical practice and usually presents no functional sequelae [94]. On the other hand, acute hypotonia can cause structural changes, depending on various factors such as the degree of hypotony, its duration and individual eye conditions, although most of the time it is anatomically and functionally reversible [95]. 

Currently, the only possible way to induce transient ocular hypotony is by performing a paracentesis. Paracentesis is widely used in ophthalmic microsurgery [96] and may also be performed at the slit lamp as an outpatient procedure. Some authors have shown the feasibility and safety of performing an immediate anterior chamber paracentesis, combined with antiglaucomatous drugs, to relief the symptoms of acute primary angle-closure glaucoma [97,98,99,100].

Induced ocular hypotony could only be considered if the paracentesis could be performed in a controlled way, with a minimal duration and extent. The leakage of aqueous through corneal paracentesis should be quick enough to decrease the intraocular pressure without causing the anterior chamber to collapse. 

The only justification to perform a paracentesis to induce a transitory ocular hypotony, causing the breakdown of the blood-ocular barrier, would be the availability of an effective drug that would reach long-term therapeutic levels, requiring maybe one or two procedures per year. Drugs with a short life could not be used in this scenario, since repetitive paracentesis could be associated with an unacceptable risk of infection and other potential complications. 

Controlled experiments investigating hypotony are scarce. Nevertheless, the occurrence of the breakdown of the ocular-blood barrier could be achieved by the induction of an iatrogenic, surgically controlled hypotony. Thus, the hypotony became a target ocular pressure through an induced condition to allow the opening of the natural vascular fenestrations into the eye. The risk of infection from a paracentesis is possibly lower than that of an intravitreal injection that penetrates into the back of the eye. However, this invasive procedure limits its use, unless one or few intravascular injections are enough to achieve the intended therapeutic response. 

Concomitantly, there are no current available hypotensive topical drugs that permit a potent decrease of the intraocular pressure in order to avoid a surgical procedure. Furthermore, the available systemic hypotensive drugs are nonselective and the aggressive side effects limit their current use. A model of chronic hypotony in an animal model has already been described [101]. The development of models of acute hypotony, associated with rigorous pharmacokinetic and anatomopathological studies, could clarify our questions about the viability of this hypothesis. Further studies on the induction of acute hypotony are necessary to establish the safety of the method, although the several studies on transient ocular hypotony have shown that, if there are no associated comorbidities, the ocular anatomy and functions are gradually normalized from the point in time that the intraocular pressure is corrected [102].

### 6.3. Inflammatory Mediators

Inflammatory mediators have been studied mainly in experimental uveitis models [103,104,105,106,107], which cause the breakdown of the blood-ocular barrier. Several inflammatory mediators have been identified during intraocular inflammation, such as prostaglandins, leukotrienes and hydroxieicosatetraenoic acids, all chemical mediators from the arachidonic acid cascade. Other identified active mediators of the immune and inflammatory response include platelet-activating factor, interleukin-1, and tumor necrosis factor [108,109], while others still remain unidentified.

Foxman *et al*. have demonstrated the inflammatory mediators during T-cell mediated ocular inflammation, such as chemokines and their receptors and cytokines, besides indicating the cytokines as potential therapeutic targets [110]. The accumulation of these mediators increases the vascular permeability, leading to the breakdown of the blood-ocular barrier and to the consequent onset of pathophysiological responses [82]. Inflammatory mediators such as the tumor necrosis factor-alpha (TNFα) have been identified as an underlying factor causing the late blood-retinal barrier breakdown in diabetic retinopathy [111]. Huang *et al*. have shown that TNFα could be a therapeutic target for the prevention of the progressive blood-retinal barrier breakdown, retinal leukostasis, and apoptosis associated with diabetic retinopathy [112]. Although some authors have demonstrated the capacity of inflammatory mediators to induce acute inflammatory reactions *in vivo*, few studies have evaluated the morphological and functional responses to inflammatory mediators in the eye [113]. 

Interestingly, ocular inflammation has also been experimentally induced through the use of synthetic mediators. The complement component C5a, *N*-formylmethionyl-leucyl-phenylananine (FMLP) and chemotactic mediators were injected into the rabbit cornea, vitreous, and skin in order to induce a reaction resembling the “Arthus phenomenon” [114]. Miyake *et al*. have demonstrated that topically used epinephrine can induce the partial disruption of the blood-ocular barrier due to the release of prostaglandins and other cyclooxygenase products [115]. Elliot *et al*. have demonstrated that bradykinin agonist RMP-7 could, experimentally, enhance the permeability of the blood-ocular barrier [116]. This class of drug may be used as a useful adjunct to achieve an enhanced delivery of therapeutics to the eye under conditions in which blood-ocular barriers limit treatment.

Inflammatory mediators are not the only way to promote the breakdown of the blood-ocular barrier. Puerarin is an isoflavonoid derived from *Radix puerariae* that has been investigated as a self-microemulsifying drug system. Deng *et al*. have shown that puerarin can penetrate the blood-ocular barrier accessing aqueous humor and vitreous humor after systemic administration [117]. This pharmacokinetic study can provide a principle basis for treating eye diseases with puerarin by systemic administration.

In the same way, recent studies have also demonstrated the possibility of rapid suppression of ocular inflammation, leading to restoration of the blood-ocular barrier. Copland *et al*. have shown that Fingolimod (FTY720), a drug used in FDA phase III human multiple sclerosis trials, prevents T-cell migration to inflammatory sites by decreasing expression of the sphingosine-1 phosphate receptor generally required for egression from secondary lymphoid tissue [118]. Fingolimod seems to be a promising anti-inflammatory drug for the treatment of ocular immune-mediated inflammation, where even a single-dose treatment was effective, and repeated doses did not affect the vascular integrity of the blood-ocular barrier [118]. Thus, the induction of a transient breakdown of the blood-ocular barrier could be immediately followed by its reversal, as soon as the intended therapeutic effect is achieved.

Therefore, the breakdown of the blood-ocular barrier could also be obtained by deliberate utilization of inflammatory mediators. However, the deliberate use of specific inflammatory mediators would only be justified based on this hypothesis, if the topical route was utilized to promote the transitory breakdown of the blood-ocular barrier. After the topical application of the inflammatory drug, the systemic administration of the specific nanosystem would be held, followed by the topical pharmacological neutralization of the inflammatory mediator previously applied, as soon as the delivery rate time of the systemic drug was concluded in order to yield therapeutic drug levels in ocular tissues.

### 6.4. Measuring the Blood-Ocular Barrier Breakdown

When the aqueous-blood barrier is broken down, there is an inward movement of plasma constituents and cells to the anterior chamber. Since 1988, it is possible to quantify, *in vivo*, the aqueous flare by measuring the scatter of a laser beam that is scanned into the anterior chamber. This reproducible and noninvasive procedure designed to measure the integrity of the blood-aqueous barrier is performed with a flare meter and is called tyndallometry [119,120,121,122,123,124]. 

Several methods of examination are currently used to evaluate the function of the blood-retinal barrier [125]. Vitreous fluorophotometry [126,127] is a technique to quantify blood-retinal barrier permeability. Optical coherence tomography is a widespread method that allows good quality image acquisition in order to evaluate blood-ocular barriers [128]. Fluorescein angiography is method to obtain excellent qualitative images of the retina, but carries some restrictions because iodinated contrast may cause severe allergic reactions and needs to be applied by an intravenous injection [129]. Contrast-enhanced magnetic-imaging is a modern and sophisticated method to measure the aqueous and vitreous humor protein concentration due to vascular leakage [130,131], but also requires an intravenous administration of gadolinium diethylenetriaminepentaacetic acid (Gd-DTPA) or other contrast agents, which limits its use in everyday practice. A recent development, the Retinal Leakage Analyzer [132], maps blood-retinal barrier changes by a multimodal macula mapping, and probably will offer an interesting additional tool to register the blood-ocular barrier dynamics. 

Thus, according to our proposition, the breakdown of the blood-ocular barrier could be monitored by the quantification of the aqueous flare and/or vitreous flare, which would help to indicate the correct time of the administration of the desired systemic medication.

### 6.5. Future Perspectives of the Possibility of Taking Advantage of Blood-Ocular Barrier Breakdown to Access Intraocular Tissues

**Scheme 3:**
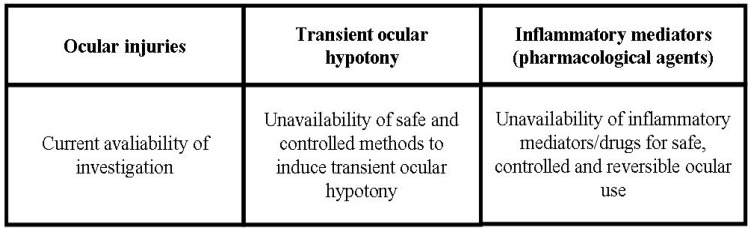
Status of investigaton of the natural and induced ways to the breakdown of blood-ocular barriers.

In summary, there are several variables that can occur in the three types of breakdown of ocular barriers, namely, in eye diseases or ocular surgeries—the intensity of inflammatory reaction; in ocular hypotony—its intensity and duration; and in the use of inflammatory mediators—the dose and specific pharmacological characteristics of the drugs. Thus, it is not currently possible to designate a preferred potential method of breakdown of the blood-ocular barrier, since the intensity and duration of the breakdown of the blood-ocular barrier are parameters highly dynamic due to the wide range of pathophysiological variables. The current absence of reliable and safe methods to induce the rupture of the blood-ocular barrier is also another important concern to establish dependable means to achieve this hypothetical goal. The current status of use or investigation of each types of breakdown of ocular barriers is summarized in Scheme 3. 

## 7. Conclusions

The proposition presented in this article refers to the possibility of taking advantage of the breakdown of the blood-ocular barrier to use the systemic route as an alternative to access intraocular tissues. Monitoring this breakdown is currently possible, indicating the correct time to use intravascular medication. Besides the natural enhanced permeability of the blood-ocular barrier in ophthalmic surgeries, we suggest two possible ways to induce a transient, controlled breakdown of the blood-ocular barrier, both with limitations that need to be overcame.

**Scheme 4:**
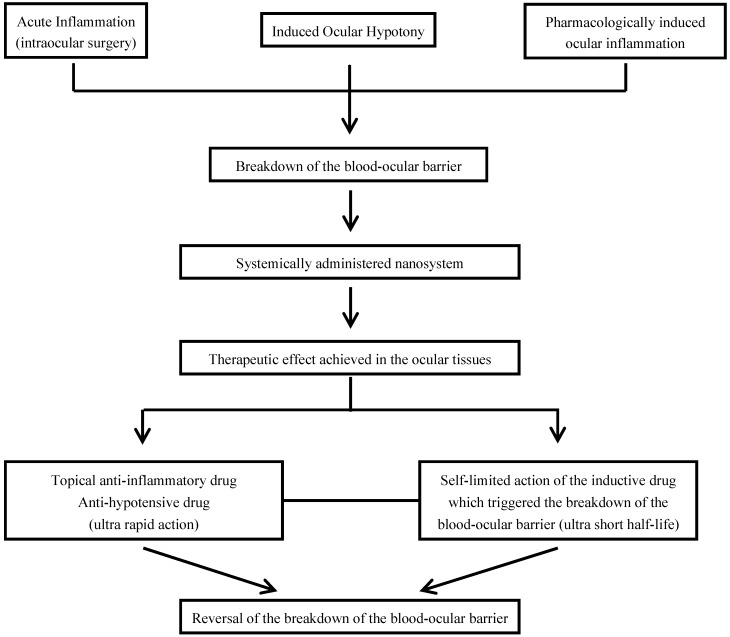
Hypothetical ways that are taken advantage of the breakdown of the blood-ocular barrier for systemically administered nanosystems.

The induced breakdown of the blood-ocular barrier would be an interesting and acceptable option to facilitate the ocular access of the systemically administered nanosystem, if a topical route is chosen to administer pharmacological agents that induce it, and likewise, other topical agents are used to reverse it after the therapeutic effect is achieved. Apart from the invasive fact of the intravenous administration of the nanosystem, only non-invasive additional topical methods would be plausible to be applied concurrently. Moreover, the reversion of the breakdown of the blood-ocular barrier, when induced, would be achieved with the use of topical anti-inflammatory or anti-hypotensive drugs. The utilization of short half-life inductive drugs would be essential to carry out a self-limiting process. Scheme 4 summarizes the hypothetical pathways that are taken advantage of the breakdown of the blood-ocular barrier in order to allow the systemically administered nanosystems to reach the ocular tissues.

The pharmacological evaluation of drugs injected into the bloodstream aiming to permeate the intraocular tissues is necessary to establish their safety, especially if formulated with nanocarriers that still require additional pharmacodynamic and pharmacokinetic studies [66,133]. The future development of methods enabling a better understanding of the complete physicochemical properties of the nanomaterials and the availability of new nanotoxicological standards will allow the real evolution in this field, making it feasible to use nanosystems via systemic route for ocular drug delivery.

In summary, the availability of new effective and safe ocular delivery systems remains a major challenge for pharmaceutical researchers. The development of methods to induce a controlled, safe and transient breakdown of the blood-ocular barrier could be a novel approach to allow the access of systemically injected drugs to the internal parts of the human eye. The ideal nanoparticle system for systemic use in order to reach the intraocular tissues is not presently available, nor is there any evidence of the facilitation of access of these nanosystems with the transient breakdown of blood-ocular barriers. However, previous studies and pathophysiological events mentioned in this article encourage us to propose this hypothesis with the aim to conceive the basis for the possible development of a viable system, allying nanoparticles properties with the facilitated permeation to intraocular tissues by taking advantage of a natural or induced breakdown of the blood-ocular barrier.

The intravenous route could be reconsidered as a good alternative to complement the ophthalmic therapeutics currently in use. As the ability to deliver drugs and other compounds across the blood-ocular barrier via the systemic route for therapeutic purposes is not reached, further studies using nanoengineered technologies and focusing on the development of nanosystems with minimal toxicity and enhanced efficacy are necessary in order to expand the knowledge of the ways to selectively and efficiently overcome the blood-ocular barrier. Moreover, more detailed studies on the normal physiological and pathological conditions of the eye are essential to yield a consistent understanding of nanosystem characteristics under different circumstances in the target organ. In addition, methods to take advantage of the natural or induced breakdown of blood-ocular barriers can complement the nanotechnological properties to get easier access to nanomedicines administered systemically and targeted to the hitherto almost impervious areas of the human eye.
